# Immortalized cells and one oncogene in malignant transformation: old insights on new explanation

**DOI:** 10.1186/1471-2121-12-23

**Published:** 2011-05-23

**Authors:** Vadym M Kavsan, Anton V Iershov, Olena V Balynska

**Affiliations:** 1 Department of Biosynthesis of Nucleic Acids, Institute of Molecular Biology and Genetics, National Academy of Sciences of Ukraine, 150 Zabolotnogo Street, 03680 Kyiv, Ukraine

## Abstract

**Background:**

Nearly thirty years ago, it was first shown that malignant transformation with single oncogene necessarily requires the immortal state of the cell. From that time this thesis for the cells of human origin was not disproved. The basic point which we want to focus on by this short communication is the correct interpretation of the results obtained on the widely used human embryonic kidney 293 (HEK293) cells.

**Results:**

Intensive literature analysis revealed an increasing number of recent studies discovering new oncogenes with non-overlapping functions. Since the 1970s, dozens of oncogenes have been identified in human cancer. Cultured cell lines are often used as model systems in these experiments. In some investigations the results obtained on such cells are interpreted by the authors as a malignant transformation of normal animal or even normal human cells (as for example with HEK293 cells). However, when a cell line gains the ability to undergo continuous cell division, the cells are not normal any more, they are immortalized cells. Nevertheless, the authors consider these cells as normal human ones, what is basically incorrect. Moreover, it was early demonstrated that the widely used human embryonic kidney 293 (HEK293) cells have a relationship to neurons.

**Conclusions:**

Thus, the experiments with established cell lines reinforce the notion that immortality is an essential requirement for malignant transformation that cooperates with other oncogenic changes to program the neoplastic state and substances under such investigation should be interpreted as factors which do not malignantly transform normal cells alone, but possess the ability to enhance the tumorigenic potential of already immortalized cells.

## 

In 1983, Newbold and Overell showed that the acquisition of the immortal state was required, although not sufficient, for malignant transformation, thus proving multistage model of cancer [1]. They had demonstrated that an oncogene could cause malignant transformation of immortalized NIH/3T3 cells. Further, many research groups demonstrated that 2 oncogenes being introduced simultaneously can cooperate in driving normal rodent cells to neoplastic state, in these pairs one of oncogenes induces immortality [2]. However, the transformation of primary human cells by these oncogene pairs were unsuccessful because of the differences in telomere biology and regulation of cell senescence between human and murine cells. As it was reviewed by Hahn, 2002 [3], "these experiments reinforce the notion that immortality is an essential requirement for transformation that cooperates with other oncogenic changes to program the neoplastic state". In the June 2010 issue of BMC Cell Biology, volume 11 in the article by Ha et al. [4] the authors undertook their study with the aim to assess whether human cervical cancer oncogene (*HCCR-1*) overexpression alone converts normal human cells to malignantly transformed cells. For this study, the authors have chosen the human embryonic kidney 293 cells (also often referred to as 293 cells, HEK 293, or less precisely HEK cells) as a model of normal human cells. In conclusion, the authors wrote: *"*although researchers have been able to transform normal mouse cells into tumor forming cells by introducing several cooperating oncogenes into these cells, human cells have been resistant to such transformation. In this study, we converted normal cells into tumor cells by delivering *HCCR-1 *alone in combination with no other oncogenes".

However, as it is widely known, 293 cells were generated by Graham et al. [5] in early 70s by transformation of culture of normal human embryonic kidney cells with sheared human adenovirus type 5 DNA. Further genomic analysis showed that approximately 4.5 kilobases from the left arm of the viral genome became incorporated into human chromosome 19, resulting in decreased senescence of these cells [6]. Thus, HEK293 is not a model for normal human cells; these cells are immortalized already by known oncogene but not malignant yet. Moreover, even NIH/3T3 cells, which authors [4] also used for their conclusions are not really normal mouse cells: NIH/3T3 cells originate from mouse embryonic fibroblasts (MEF), which have the ability to overcome cellular senescence and be spontaneously immortalized when cultured under certain conditions [7]. The mechanism of this immortalization was not fully described, but it was proposed that *p19ARF *and *MDM2 *functionally inactivate both p53 and pRb pathways involved in cell senescence [8]. NIH/3T3 can be oncogenically transformed by Ras alone [9], while for MEF transformation at least 2 oncogenes are necessary [2].

Interesting, that similar investigations on HEK293 or NIH/3T3 cell lines with other oncogenes which do not overlap in their intracellular function also show conversion into tumor cells with one oncogene alone [4,10-12]. It is quite difficult also to accept completely the interpretation of authors that they observed transdifferentiation in HEK293 cell line without further discussion. The matter is that previously Shaw et al. [13] demonstrated that *"*the widely used HEK293 cells have an unexpected relationship to neurons, a finding that may require reinterpretation of many previous studies in which it was assumed that HEK293 cells resembled more typical kidney epithelial cells" and may not be used as an *in vitro *model for kidney cell function.

Thus, the experiments with an established or immortalized cell line that has acquired the ability to proliferate indefinitely either through random mutation or deliberate modification, such as ectopic expression of different genes, reinforce the notion that immortality is an essential requirement for transformation that cooperates with other oncogenic changes to program the neoplastic state and that the substances under such kind of investigations should be interpreted as factors (in particular, oncogenes or oncoproteins) which not malignantly transform alone the normal cells, but possess the ability to enhance the tumorigenic potential of already immortalized cells.

Nevertheless, the article by Ha et al. [4] was published very timely because the findings about the origin of HEK293 and NIH/3T3 cell lines will require reinterpretation of many previous studies in which it was assumed that these and other immortalized cell lines resembled typical normal human or rodent cells.

## Authors' contributions

All the authors contributed equally to the text.

## References

[B1] Newbold RF Overell RW Fibroblast immortality is a prerequisite for transformation by EJ c-Ha-ras oncogene Nature 1983 304 648 651 10.1038/304648a0 6877385

[B2] Land H Parada LF Weinberg RA Tumorigenic conversion of primary embryo fibroblasts requires at least two cooperating oncogenes Nature 1983 304 596 602 10.1038/304596a0 6308472

[B3] Hahn WC Immortalization and transformation of human cells Mol Cells 2002 13 351 361 12132573

[B4] Ha SA Kim HK Yoo J Kim S Shin SM Lee YS Hur SY Kim YW Kim TE Chung YJ Transdifferentiation-inducing HCCR-1 oncogene BMC Cell Biol 2010 11 49 10.1186/1471-2121-11-49 20591135 PMC2909153

[B5] Graham FL Smiley J Russell WC Nairn R Characteristics of a human cell line transformed by DNA from human adenovirus type 5 J Gen Virol 1977 36 59 74 10.1099/0022-1317-36-1-59 886304

[B6] Louis N Evelegh C Graham FL Cloning and sequencing of the cellular-viral junctions from the human adenovirus type 5 transformed 293 cell line Virology 1997 233 423 429 10.1006/viro.1997.8597 9217065

[B7] Todaro GJ Green H Quantitative studies of the growth of mouse embryo cells in culture and their development into established lines J Cell Biol 1963 17 299 313 10.1083/jcb.17.2.299 13985244 PMC2106200

[B8] Carnero A Hudson JD Price CM Beach DH p16INK4A and p19ARF act in overlapping pathways in cellular immortalization Nat Cell Biol 2000 2 148 155 10.1038/35004020 10707085

[B9] Thorgeirsson UP Turpeenniemi-Hujanen T Williams JE Westin EH Heilman CA Talmadge JE Liotta LA NIH/3T3 cells transfected with human tumor DNA containing activated ras oncogenes express the metastatic phenotype in nude mice Mol Cell Biol 1985 5 259 262 3982418 10.1128/mcb.5.1.259-262.1985 PMC366702

[B10] Wang YL Wang Y Tong L Wei Q Overexpression of calcineurin B subunit (CnB) enhances the oncogenic potential of HEK293 cells Cancer Sci 2008 99 1100 1108 10.1111/j.1349-7006.2008.00799.x 18422742 PMC11158933

[B11] Lin YL Han ZB Xiong FY Tian LY Wu XJ Xue SW Zhou YR Deng JX Chen HX Malignant transformation of 293 cells induced by ectopic expression of human Nanog Mol Cell Biochem 2011 351 109 116 10.1007/s11010-011-0717-5 21246261

[B12] Hamid T Malik MT Kakar SS Ectopic expression of PTTG1/securin promotes tumorigenesis in human embryonic kidney cells Mol Cancer 2005 4 3 10.1186/1476-4598-4-3 15649325 PMC546418

[B13] Shaw G Morse S Ararat M Graham FL Preferential transformation of human neuronal cells by human adenoviruses and the origin of HEK 293 cells FASEB J 2002 16 869 871 11967234 10.1096/fj.01-0995fje

